# The Interplay of Light Signaling and *SlAN1* Expression in Regulating Anthocyanin Accumulation in Fruit Tissues of Purple Tomatoes

**DOI:** 10.1111/ppl.70740

**Published:** 2026-01-22

**Authors:** Gabriel Lasmar dos Reis, Chaiane Fernandes Vaz, Luis Willian Pacheco Arge, Adolfo Luís dos Santos, Samuel Chaves‐Silva, Agustín Zsögön, Lázaro Eustáquio Pereira Peres, Antonio Chalfun‐Junior, Vagner Augusto Benedito

**Affiliations:** ^1^ School of Agriculture and Food Systems West Virginia University Morgantown West Virginia USA; ^2^ Department of Biology Universidade Federal de Lavras (UFLA) Lavras Brazil; ^3^ Department of Agronomy and Plant Genetics University of Minnesota Saint Paul Minnesota USA; ^4^ National Institute of Science and Technology on Plant Physiology Under Stress Conditions, Department of Plant Biology Universidade Federal de Viçosa Viçosa Brazil; ^5^ Laboratory of Hormonal Control of Plant Development, Luiz de Queiroz College of Agriculture, Department of Biological Sciences University of São Paulo Piracicaba Brazil; ^6^ School of Agricultural and Natural Sciences University of Maryland Eastern Shore Princess Anne Maryland USA

**Keywords:** antioxidants, crop improvement, food and health, natural dye, natural genetic variation, near‐isogenic line (NIL)

## Abstract

Anthocyanins are specialized plant metabolites with significant dietary value due to their anti‐inflammatory properties. Research indicates that dietary intake of these phenolic compounds contributes to preventing various chronic diseases. As the most consumed vegetable worldwide, tomato ( *Solanum lycopersicum* ) is an excellent candidate for anthocyanin‐enrichment strategies. In tomato, the activation of anthocyanin biosynthesis is light‐dependent, but this mechanism has yet to be entirely characterized. We investigated the role of light in anthocyanin biosynthesis in purple tomato fruits generated by combining the *Anthocyanin fruit* (*Aft*), *atroviolacea* (*atv*), and *high‐pigment 2* (*hp2*) mutations into cv. Micro‐Tom (MT). MT‐*Aft/atv/hp2* starts accumulating anthocyanins early during fruit development, but this accumulation is restricted to the peel (exocarp and epicarp). By manipulating light incidence in different fruit tissues, we determined that the absence of anthocyanin accumulation in the flesh results from the sun‐blocking effect of the cyanic epicarp on the flesh (mesocarp), thus preventing light from penetrating deeper into the fruits. Comparative transcriptional analyses of the fruit peel and flesh indicated that the bHLH transcription factor SlAN1 (*Solyc09g065100*) may be the limiting factor for light‐dependent anthocyanin accumulation in both tissues. This research enhances our comprehension of the genetic and environmental regulation of anthocyanin accumulation in fruit tissues, offering valuable insights into plant breeding for human nutrition.

## 1 Introduction

Anthocyanins are natural pigments derived from the plant's specialized metabolism that confer red, pink, purple, or blue pigmentation to plant tissues, depending on the molecular structure and vacuolar pH for their final hue (Hichri et al. 2010; Houghton et al. 2021). Beyond the ecological notion that anthocyanins are responsible for attracting pollinators and seed dispersers, they also play a protective role due to their antioxidant activity by scavenging reactive oxygen species (ROS) that otherwise could severely damage plant tissues (Buer et al. 2010; Corso et al. 2020). Furthermore, it has been suggested that anthocyanins form a protective barrier in plant tissues by absorbing UV‐B radiation potentially harmful to the photosynthetic machinery (Gould et al. 2010; Cerqueira et al. 2023). These properties make anthocyanins an important protective compound against environmental stresses. From a dietary perspective, based on their antioxidant and anti‐inflammatory properties, anthocyanins are bioactive in preventing or mitigating a series of chronic diseases (Martin et al. 2011; Panchal et al. 2022), such as cardiovascular disorders (Cassidy et al. 2013), type 2 diabetes (Fallah et al. 2020), obesity (Muraki et al. 2013), and cancer (Butelli et al. 2008). Therefore, they represent an important health‐promoting compound that should be consistently incorporated into the diet.

Considering the nutritional value of horticultural crops in the human diet as sources of antioxidants, studies were carried out on these species to understand the regulatory mechanisms of the anthocyanin biosynthesis pathway (for a review, cf. Chaves‐Silva et al. 2018). In this context, anthocyanin‐enriched (cyanic) versions of fresh produce represent a critical source of anthocyanins that is readily available and cost‐effective, allowing dietary enrichment simply by choosing cyanic varieties. Anthocyanins are typically found in limited quantities in cyanic products because they are often confined to the epidermal and subepidermal cells (epicarp, exocarp, or peel), which account for only 3%–5% of the fruit's total mass (Sestari et al. 2014; Chaves‐Silva et al. 2018). Thus, devising breeding strategies to generate anthocyanin‐enriched varieties with an emphasis on the mesocarp (flesh) of horticultural crops is critical.

Worldwide, tomato ( *Solanum lycopersicum* ) is the most consumed vegetable. It is thus an excellent model for discovering strategies aiming at anthocyanin enrichment. Although the fruits of most cultivated varieties of tomato do not accumulate anthocyanins (Povero et al. 2011; Sestari et al. 2014), some related wild species, such as *S. lycopersicoides* , *S. peruvianum* , and *S. chilense* , accumulate small amounts in the subepidermal layers under adequate light conditions (Bedinger et al. 2011; Chaves‐Silva et al. 2018).

Traditional breeding has delivered some varieties with cyanic tomato fruits. The introgression of the alleles *Anthocyanin fruit* (*Aft*) and *atroviolacea* (*atv*) from *S. chilense* and *S. cheesmaniae* , respectively, into *S. lycopersicum* led to purple tomato varieties, such as the cv. Indigo Rose, with an epicarp with high levels of anthocyanins (Mes et al. 2008; Gonzali et al. 2009). The further stacking of the mutation *high pigment 2* (*hp2*) from cv. Manapal (Mustilli et al. 1999), which confers hypersensitivity to light‐mediated responses, into the double mutant in the cv. Micro‐Tom background led to a genotype (MT‐*Aft/atv/hp2*) with very high anthocyanin content in the epicarp (Sestari et al. 2014), but low accumulation in the mesocarp (Gonzali et al. 2025). The *hp2* allele corresponds to a loss‐of‐function mutation in the gene *DEETIOLATED1* (*DET1*), a negative regulator of photomorphogenesis and anthocyanin biosynthesis that acts via COP1‐mediated degradation of the transcription factor HY5 (Menconi et al. 2025). In *hp2* mutants, the absence of functional DET1 stabilizes HY5, promoting pigment accumulation even under heat stress conditions (Menconi et al. 2025). Nevertheless, the proposed impairment of COP1 activity in DET1 mutants has been demonstrated only for HY5 and warrants cautious interpretation. While HY5 may evade degradation in *hp2* genotypes, COP1 may retain activity toward other substrates.

The anthocyanin biosynthesis pathway is controlled by the action of specific transcription factors (TFs), which are influenced by plant development and environmental stimuli (Albert et al. 2014). Among these, R2R3 MYBs can act individually or together with bHLH and WDR TFs in a multiprotein complex (MBW). On the other hand, R3 MYB is a competitive inhibitor of the anthocyanin biosynthesis pathway mediated by the MBW. This complex controls the expression of the structural genes, the “early biosynthetic genes” (EBGs) and “late biosynthetic genes” (LBGs), which code for enzymes essential to anthocyanin biosynthesis in different tissues (Chaves‐Silva et al. 2018; Colanero, Perata, and Gonzali 2020). In tomato, ATV is an R3 MYB, while AFT is an R2R3 MYB. This is consistent with the observation that the recessive *atv* allele, characterized by a loss‐of‐function due to a premature stop codon, and the dominant *Aft* allele, both contribute to the enhancement of anthocyanin biosynthesis (Cao et al. 2017; Colanero et al. 2018; Colanero, Tagliani, et al. 2020).

Light is one of the most critical environmental factors that control anthocyanin biosynthesis (Albert et al. 2009). Shading or dark conditions repress the expression of structural genes in this pathway (Hong et al. 2015; Liu et al. 2020). In some tomato genotypes (e.g., cv. “Indigo Rose” and *Aft/Aft*: LA1996), the expression of anthocyanin‐related genes in the epicarp starts at the early green stage and is influenced by the amount of light received on each side of the fruit, with the shaded side exhibiting a green hue compared to a darker purple hue on the side exposed to direct light (Qiu et al. 2019; Colanero, Tagliani, et al. 2020).

A critical unresolved question is the mechanism behind internal parenchymatic tissues often being less prone to accumulate anthocyanins than epidermal tissues in plants (Chaves‐Silva et al. 2018). Here, we investigated how light influences anthocyanin accumulation in purple fruit tissues of the tomato genotype MT‐*Aft/atv/hp2* by blocking light during fruit development. We explored global transcriptional activity in response to light in dissected tissues of the fruit. This work sheds light on the transcriptional regulation of anthocyanin pigmentation in response to light. Our study also provides new insights for achieving higher anthocyanin content in fleshy fruits.

## 2 Materials and Methods

### 2.1 Plant Material, Growth Conditions, and Sampling

To characterize the starting point of anthocyanin pigmentation in tomato fruits, we used the cyanic genotype (MT‐*Aft/atv/hp2*; Sestari et al. 2014) and the cultivar Micro‐Tom (Meissner et al. 1997) as a control. The plants were grown in a greenhouse under a 16 h photoperiod with 600–700 W/m^2^ radiation, 21°C ± 2°C, and 50% RH. Floral buds, flowers, and developing fruits from both genotypes were collected from the plants, placed against a black background, and photographed. The resulting images were analyzed using the NIS Elements software (Nikon Instruments).

For phenotypic characterization and RNA‐seq analysis, individual flowers were covered with aluminum foil at anthesis and kept for 30 days, aiming at fruit development in the complete absence of light (Figure S1). Subsequently, the developed fruits were exposed to light and collected at different times: immediately (0d), 2 days (2d), and 5 days (5d) after the cover was removed. For a positive control treatment (Ctl), flowers were marked at anthesis, left exposed to normal light, and the fruits were collected after 30 days. All fruits collected were dissected into the epicarp (the peel or exocarp) and mesocarp (the flesh), and the seeds were discarded. All plant material was snap‐frozen in liquid nitrogen and stored in a −80°C freezer until use. Material from three plants was collected and pooled to represent a biological replicate, and three biological replicates were used in the following analyses.

For the RT‐qPCR assay, plants from MT‐*Aft/atv/hp2* were grown under natural irradiation with supplemental lighting provided as needed by high‐pressure sodium lighting (Hortilux 600 W deep reflector HS200), with a 16 h photoperiod at 21°C ± 2°C. Fruits at the mature green stage of development were collected, and the peel (epicarp) and flesh (mesocarp) were dissected. The seeds were discarded. The material was collected from three individual plants, and together, they represented one biological replicate. Three biological replicates were used in the analyses. All plant material was immediately frozen in liquid nitrogen and stored in a −80°C freezer until RNA extraction.

### 2.2 RNA Isolation, Library Preparation, and Sequencing

Total RNA was extracted from the epicarp and mesocarp separately using the mirVana miRNA isolation kit (Ambion, ThermoFisher) according to the manufacturer's instructions. The integrity of the RNA was visualized on a 1.2% agarose gel, and the quantity and quality were assessed on a Nanodrop spectrophotometer. Library preparation and Illumina sequencing were performed at Novogene (www.novogene.com). Twenty‐four cDNA libraries were prepared and then sequenced on an Illumina Novaseq 6000, with a configuration of 150‐bp paired‐end. The sequencing of transcripts revealed a total of 585.6 million reads (a mean of 23.7 million reads per library), of which 568.8 million (97%) showed sufficient quality (*Q* > 20). The libraries were aligned on the *Solanum lycopersicum* genome assembly v.4.0 and ITAG annotation v.4.2 from SolGenomics (https://solgenomics.net; Fernandez‐Pozo et al. 2015). On average, 22.2 million reads per library (94%) were uniquely mapped on the genome (Table S1).

### 2.3 Pre‐Processing and Mapping of Reads

Raw FastQ data were initially submitted for quality analysis with FastQC v.0.11.5 (Andrews 2010). The cleaning step was performed with Trimmomatic v.0.39 (Bolger et al. 2014), with the following parameters: ILLUMINACLIP:TruSeq3‐PE.fa:2:30:10:2:keepBothReads to remove adapters and keep both as paired reads, LEADING:3 and TRAILING:3 to remove bases below the quality of three at the start and final of each read, respectively, and MINLEN:36 to discard reads with < 36 bp. Each cleaned library was submitted for read mapping against the genome with the software Star v.2.7.5.c (Dobin et al. 2013). The counting of mapped reads for each gene was performed with featureCounts v.1.6.5 (Liao et al. 2014).

### 2.4 Differential Expression, Functional Annotation, and Enrichment Analysis

Genes with low expression values (TPM < 1—transcripts per million) were removed from the differential expression analysis. Differentially expressed genes were identified in R v.4.0.5 with the package DESeq2 v.1.32.0 (Love et al. 2014). All three replicates for each time point were used for the differential expression analysis, and the comparisons were performed against the same tissue. Genes with adjusted *p*‐values < 0.05 were considered differentially expressed (DEGs). The package ggplot2 v.3.3.2 (Wickham 2016) was used to build bar charts, and ComplexHeatmap v.2.9.0 (Gu et al. 2016) for heatmaps.

Tomato gene features were retrieved from different databases. Transcription factor information was retrieved from the PlantTFDB v.5.0 (Jin et al. 2016). Annotation of genes encoding metabolic enzymes was performed in the KEGG (Kyoto Encyclopedia of Genes and Genomes) database with GhostKOALA v.2.2 (Kanehisa et al. 2016). Gene Ontology (GO) terms were annotated with GOMAP v.1.3.4 (Wimalanathan and Lawrence‐Dill 2021) to obtain a high coverage level of genome annotation. The R's match function was used to retrieve functional information from the genome and match it to a set of DEGs.

To investigate the primary functional annotations of the DEGs sets in each comparison, we conducted an enrichment analysis to identify overrepresented metabolic pathways, transcription factors, and GO terms in the dataset. This approach was applied for each DE profile, separated by up‐ and down‐regulated genes. We considered features below the *p*‐value < 0.05 threshold for metabolic pathways and transcription factors, and FDR < 0.05 for GO terms as overrepresented. Due to the high number of enriched GO terms, we computed the semantic similarity between terms with the mgoSim function from the R package GOSemSim v. 2.24.0 (Yu et al. 2010; Yu 2020). Following this, a dimensional reduction analysis was conducted using the UMAP (Uniform Manifold Approximation and Projection) function from the UMAP v. 0.2.10 (McInnes et al. 2018) R package. dbscan function, package dbscan v. 1.1‐11 (Hahsler et al. 2019), was applied to identify GO term clusters using eps of 0.4 and a minimum of five points. Ggplot2 and ggConvexHull were used to plot UMAP and bubble charts, and each clustering of GO terms was labeled by the lowest FDR value.

### 2.5 Transcriptional Metabolic Flux Information Analysis

The phenotypes of the cyanic mutant tomato line were investigated using an information flux deviation analysis focused on metabolic pathways related to light and anthocyanins. We reconstructed these metabolic pathways and employed a fluxomics‐like approach based on gene expression and differential expression data to identify deviations in metabolic fluxes.

To conduct this analysis, we needed to reconstruct the metabolic pathways using the concept of gene orthology. This involved retrieving information from KEGG orthology (Kanehisa et al. 2016), identifying orthologs from Arabidopsis, using OMA (Altenhoff et al. 2024), and utilizing metabolic genes previously identified in tomato.

### 2.6 RNA Isolation, DNase Treatment, and cDNA Synthesis

Total RNA was extracted from epicarp and mesocarp separately using Trizol reagent (Invitrogen). The integrity of the RNA was visualized on a 0.8% agarose gel, and the quantity and quality (ratio 260/280 and 260/230 between 1.8 and 2.2) were measured on a Nanovue spectrophotometer. RNA samples (5.0 μg) were treated with a Turbo DNA‐free kit (Life Technologies) to eliminate residual genomic DNA. Subsequently, 1 μg of the treated RNA from each sample was submitted to cDNA synthesis using the High‐Capacity cDNA Reverse Transcription Kit (Thermo Fisher).

### 2.7 Real‐Time PCR Analysis

For the quantitative real‐time PCR (RT‐qPCR), the standard QuantiNova SYBR Green PCR Kit (QIAGEN) protocol was used. The compounds of these reactions were 1.5 μL cDNA, 1.5 μL of each primer (forward and reverse) at a final concentration of 1 μM, 7.5 μL of QuantiNova SYBR Green PCR Master Mix (QIAGEN), and 3.0 μL of water to a final reaction volume of 15 μL for each reaction. These final reactions were incubated at 95°C for 2 min, followed by 40 cycles at 95°C for 5 s and 60°C for 10 s. Then, the samples were heated from 55°C to 95°C with an increase of 1°C to acquire the melting curve of the amplified products. All reactions were run in duplicates.

The efficiency of each primer was calculated by a standard curve of a 1:5 serial dilution of a cDNA pool. Primer sequences are listed in Table S2. The normalized comparative Cq (quantitative Cycle) method was used (Pfaffl 2001) to calculate relative expression using *Solanum U6* and *5.8S* as reference genes.

### 2.8 Statistics

Statistical analyses of the RT‐qPCR data were performed using the R software (Team 2013). The normality of variables was assessed using the Shapiro–Wilk test. The *t*‐test was applied to the data. All tests were used with a significance level of 95% (*p* ≤ 0.05).

## 3 Results

### 3.1 The Onset of Anthocyanin Pigmentation in the Cyanic Tomato Fruit (MT‐*Aft/atv/hp2*)

**FIGURE 1:**
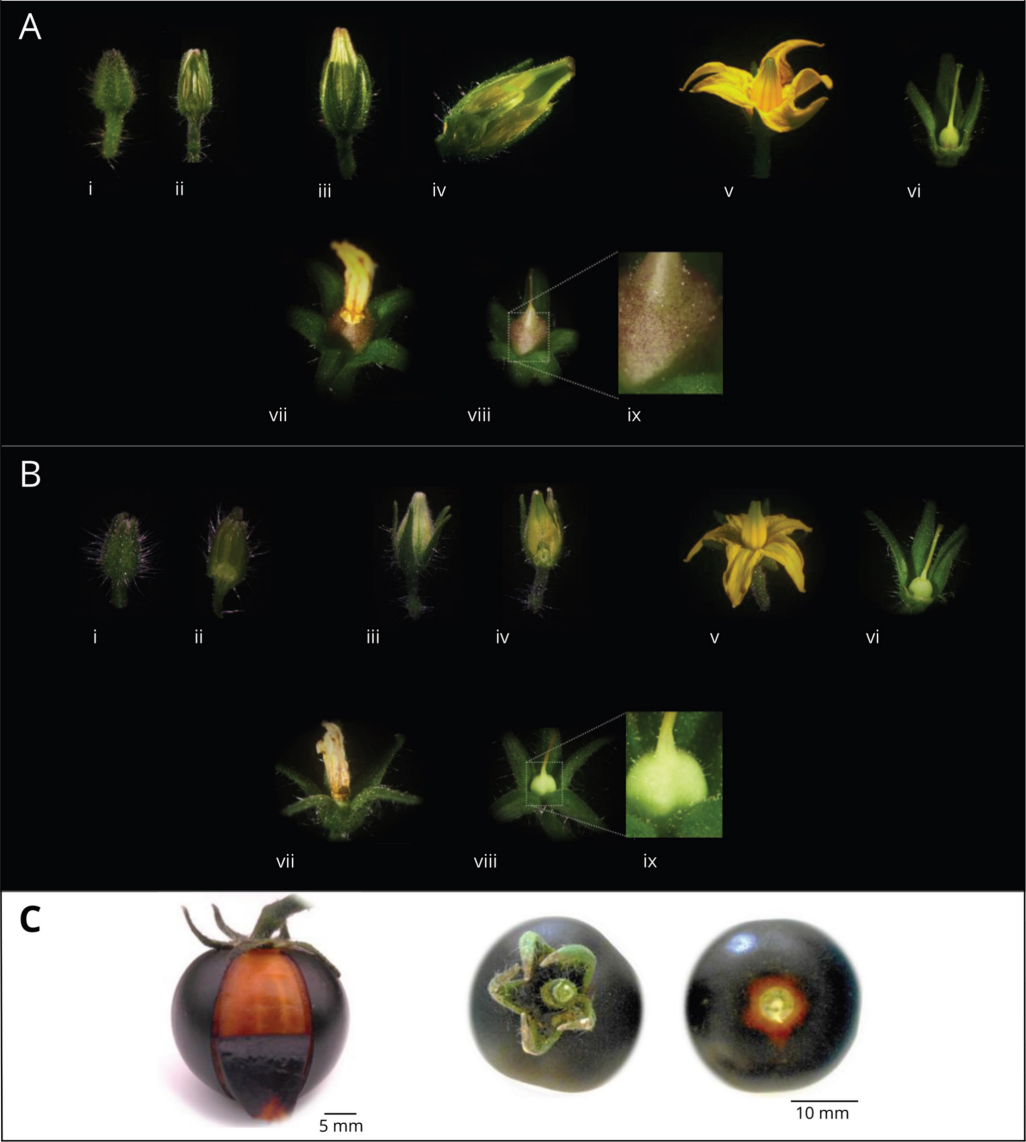
Monitoring the flowering and fruit development of two Micro‐Tom (MT) genotypes and light‐dependent anthocyanin accumulation patterns in purple tomato fruits. (A) Floral buds, flowers, and developing fruits in the purple‐fruit genotype, MT‐*Aft/atv/hp2*, and (B) the regular, red‐fruit cv. Micro‐Tom (control). (i) Developing floral bud; (ii) Cross‐section of the developing floral bud; (iii) Immature flower; (iv) Cross‐section of the immature flower; (v) Flower anthesis; (vi) Flower anthesis without the petals; (vii) Floral senescence; (viii) Fruit in early development at floral senescence; (ix) Zoom in on the early developing fruit shown in (viii). (C) Lack of anthocyanin accumulation in the mesocarp and proximal region of mature fruits (MT‐*Aft/atv/hp2*) when growing under normal light conditions. Notice the lack of anthocyanin accumulation in the epidermis under the calyx due to the lack of direct light exposure.

We monitored flowering and fruit development in MT‐*Aft/atv/hp2* plants and identified that anthocyanin accumulation starts right after flower senescence. As the petals fell off and the young fruit was directly exposed to light, anthocyanin accumulated in the epicarp (Figure 1A). In contrast, fruits of the control genotype (cv. Micro‐Tom) did not show visible anthocyanin pigmentation, as expected (Figure 1B). This pattern of anthocyanin accumulation in MT‐*Aft/atv/hp2* plants is independent of further fruit development but restricted mainly to the epicarp, while the mesocarp and the region under the sepals remained acyanic (Figure 1C).

### 3.2 Light Activates Anthocyanin Biosynthesis in the Tomato Mesocarp

**FIGURE 2:**
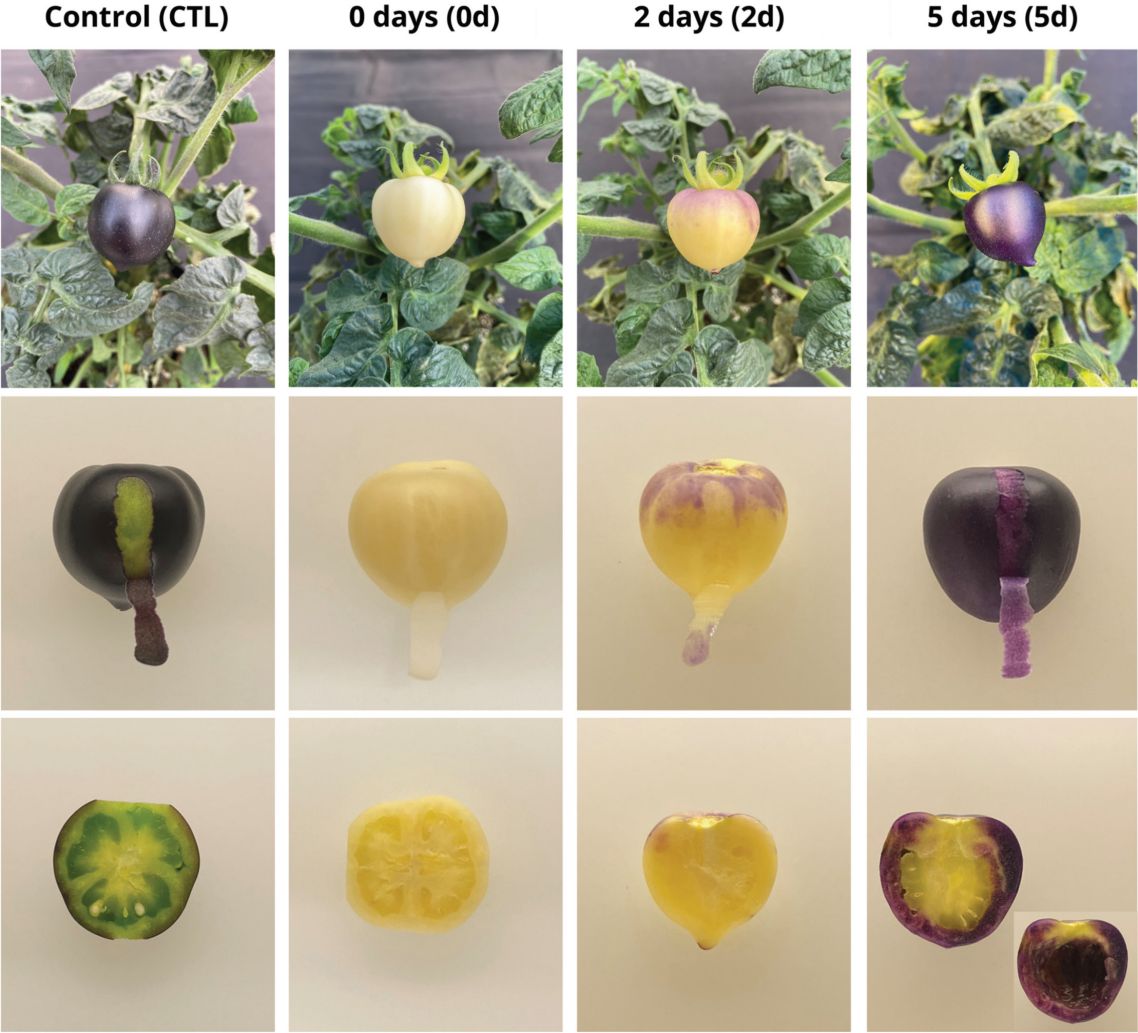
Phenotypic characterization of the anthocyanin pigmentation patterns. The epicarp and mesocarp of the cyanic tomato genotype (MT‐*Aft/atv/hp2*) developed in the dark for 30 days post‐anthesis. Tissues of fruit developed under different light conditions: Not covered (control); immediately after the removal of the foil cover (0d); 2 days (2d); and after 5 days (5d) after cover removal. The 2d and 5d fruits were cut longitudinally to better visualize the anthocyanin accumulation in the mesocarp tissue. The inset of the 5d mesocarp cross‐section displays the internal side of the mesocarp by removing the inner fruit tissues.

Based on the light‐dependent pattern of anthocyanin pigmentation in MT‐*Aft/atv/hp2* purple fruits, we investigated the metabolic response when restricting light incidence on the fruit from its first developmental stages and then exposing the mature green fruit to light. Immediately after anthesis, individual flowers were covered with aluminum foil to allow the fruit to develop for 30 days to the mature green stage in the complete absence of light (Figure S1). As a control, flowers from other plants were marked simultaneously but not covered. Compared to control fruits, which developed a dark purple phenotype in the epicarp and a green phenotype in the mesocarp (Figure 2), the covered fruits remained acyanic at the removal of the cover (0 d; Figure 2). Notably, even without light, fruits developed normally in size and shape, reaching a diameter of 3 cm.

To investigate the activation of the anthocyanin biosynthesis after the fruit was fully developed in the dark, we exposed them to normal light conditions for up to 5 days after the cover had been removed and examined the anthocyanin pigmentation phenotype in the epicarp and mesocarp. Exposure to light for 2 days (2d) showed visible signs of anthocyanin accumulation in sectors of the fruit epicarp and mesocarp (Figure 2). Furthermore, exposure to light for 5 days (5d) led to a notable increase in purple pigmentation in the entire epicarp and, surprisingly, also in the mesocarp (Figure 2). The progressive development of the purple hue in the fruits is shown in Figure S2.

### 3.3 Global Transcriptional Expression Analysis of Tomato Fruit Tissues in Response to Light

**FIGURE 3:**
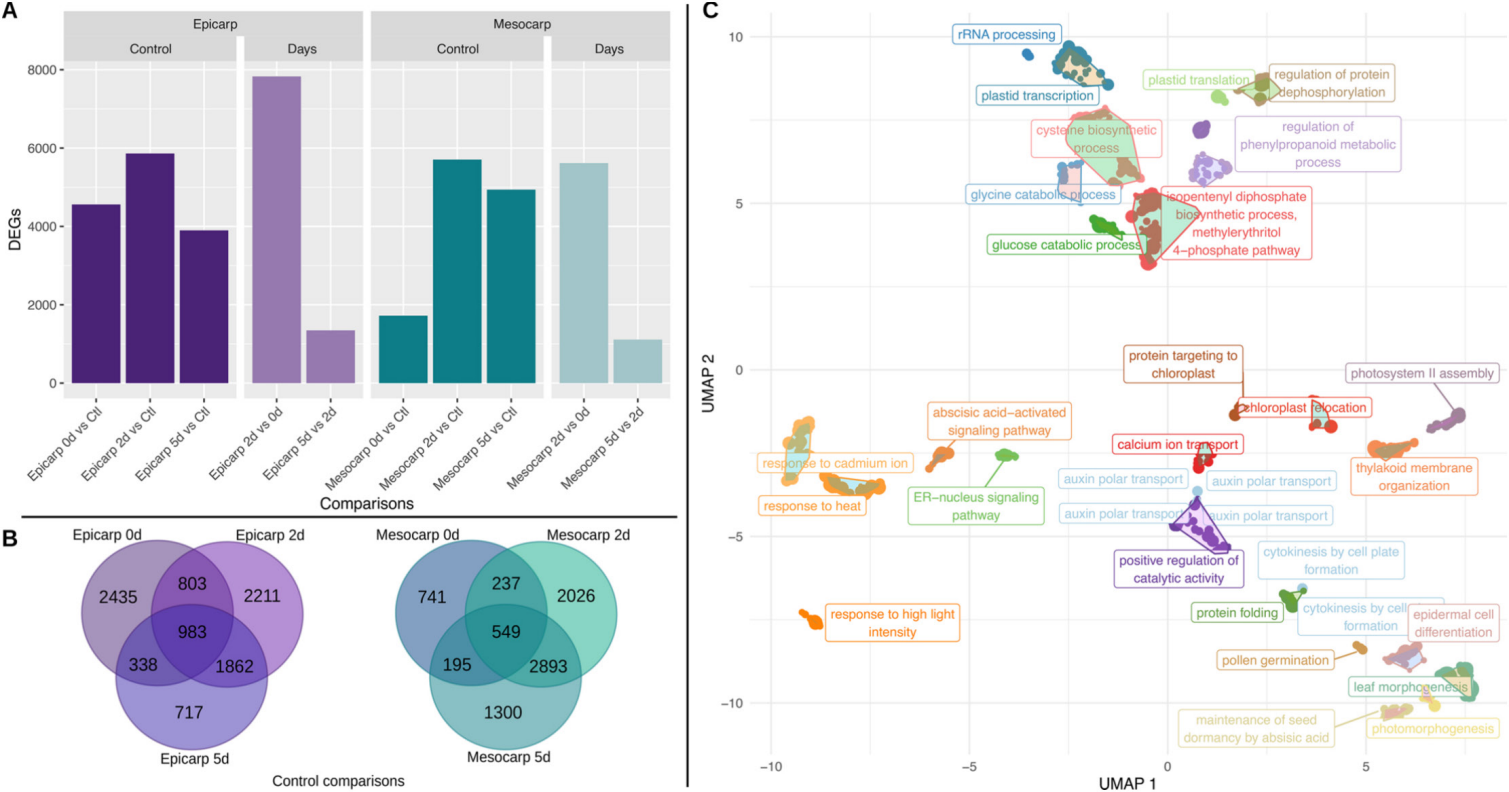
Differentially expressed genes (DEGs) and enriched GO terms in tomato fruit tissues (MT‐*Aft/atv/hp2*) in response to different light exposure conditions. (A) Total number of DEGs in different comparisons. (B) Venn diagrams for differential expression within the same tissue in different light conditions versus the control (fruits grown under normal light conditions). (C) Summary of all enriched GO terms (biological process) clustered by semantic similarity using UMAP (Uniform Manifold Approximation and Projection). Each cluster was labeled by the GO term with the lowest FDR. Abbreviations: Ctl, control (fruit grown under light conditions); d, days; Log2FC, logarithmic base 2 of fold change.

To further understand the molecular mechanisms behind the light‐mediated transcriptional regulation of anthocyanin accumulation in the tomato fruits, we carried out RNA‐seq analysis of the epicarp and mesocarp of the treatments shown in Figure 2. By comparing the epicarp of control (fruits developed under light) and fruits exposed to light for 0 (immediately after uncovering), 2, and 5 days after uncovering, we identified 4559, 5859, and 3900 DEGs, respectively. The expression of 983 genes in the epicarp differed in 0, 2, and 5 days of light exposure compared to the control. Meanwhile, we found 1722 (0d), 5705 (2d), and 4937 (5d) DEGs in the mesocarp compared to the control (Figure 3A; Table S3). In this tissue, 549 genes were identified as differentially expressed coincidently in 0, 2, and 5 days of light exposure, compared to the control (Figure 3B). The heatmap representing all DEGs is shown in Figure S4. Expression values for TPM and DEGs can be accessed in the Tables S4 and S5, respectively. This analysis revealed a light‐triggered transcriptional network involved in anthocyanin accumulation in fruit tissues.

Enrichment analyses of functional gene annotations can reveal critical transcriptional changes associated with a phenotype. Therefore, we conducted enrichment analyses of metabolic pathways, transcription factor (TF) families, and gene ontology (GO) terms to identify distinct patterns triggered by light in fruit tissues. Seventy‐five metabolic pathways were enriched in both the epicarp and mesocarp upon light exposure, including those directly or indirectly associated with anthocyanin biosynthesis and specialized metabolism: flavone and flavonol biosynthesis (map00944); flavonoid biosynthesis (map00941); phenylalanine metabolism (map00360); phenylalanine, tyrosine, and tryptophan biosynthesis (aromatic amino acids: map00400); along with the biosynthesis of carotenoids (map00906) and terpenes (map00900), and the degradation of geraniol (map00281). Aromatic amino acid biosynthesis was not enriched in the mesocarp at 0d light exposure but at 2d and 5d of light exposure when compared with the mesocarp of the control fruit developed under light conditions (Figure S5; Table S6).

Transcription factors (TFs) play critical roles in modulating gene expression. We identified 15 TF families enriched in the various DEG comparisons (Figure S5). The most noticeable family was MYB at 2d and 5d light exposure compared to the control. Other anthocyanin‐related TF families were identified, such as SQUAMOSA promoter‐binding protein‐like (SBP box) and Double B‐box (DBB) proteins (Figure S5). Further analysis of the DEG sets allowed us to identify 27 clusters of enriched GO terms associated with light‐mediated responses. A clustering of light responses was found with 20 GO terms, which include response to far‐red light (GO:0010218), response to red light (GO:0010114), response to high light intensity (GO:0009644), and the profiles with the highest number of enriched GO terms were downregulated in the mesocarp at 2d and 5d (against control). Interestingly, anthocyanin‐containing compound biosynthesis (GO:0009718) was enriched in both tissues (Figure S5) when anthocyanin accumulation became perceptible.

### 3.4 Expression of Photoreceptor Genes in the Anthocyanin Biosynthesis Pathway

**FIGURE 4:**
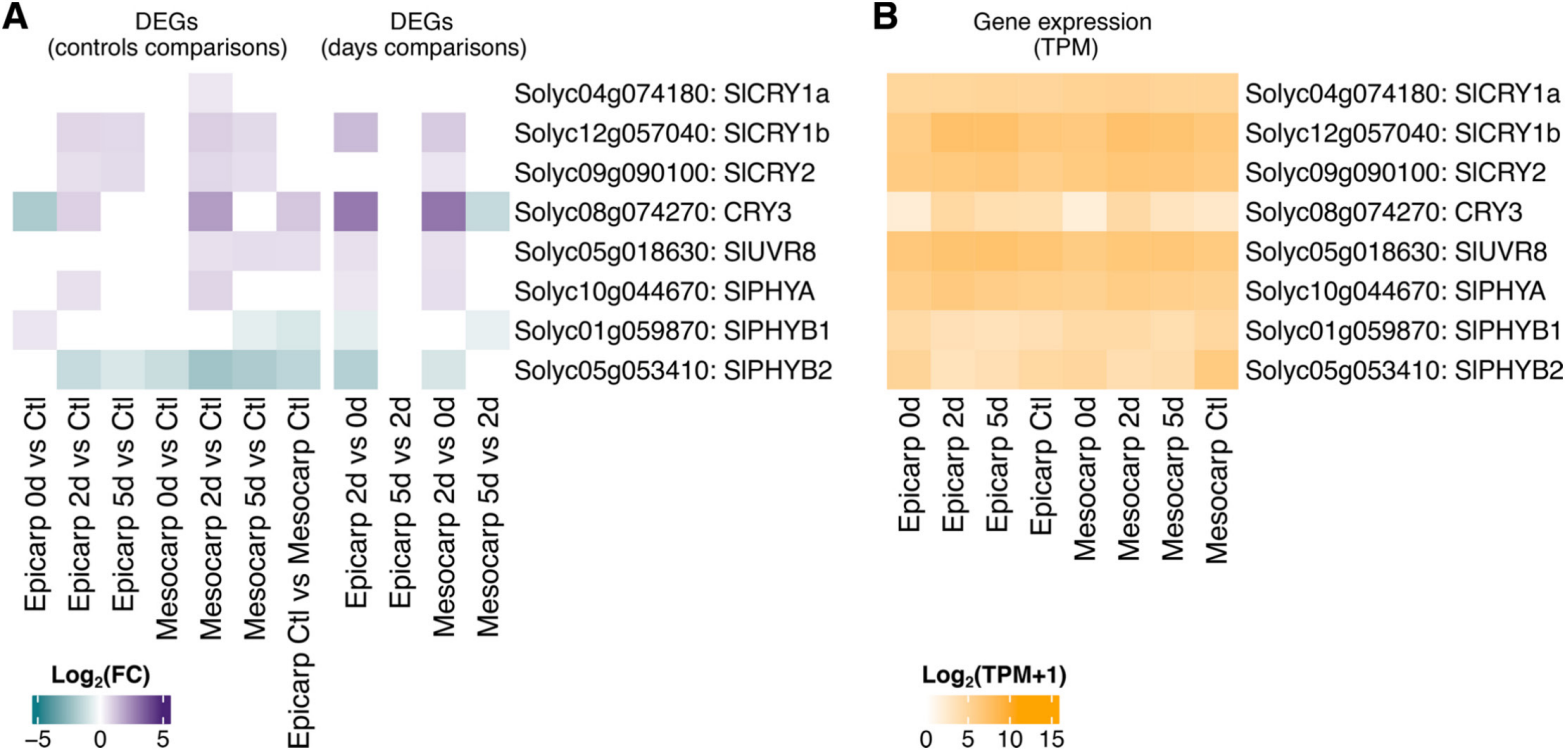
Transcriptional level of the anthocyanin photoreceptor genes in tissues of tomato fruits (MT‐*Aft/atv/hp2*). (A) differential expression (log2 FC) of the photoreceptor genes involved in the anthocyanin biosynthesis pathway. (B) Expression [log2 (TPM + 1)]. Abbreviations: Ctl, control; d, days of light exposure after cover removal; DEGs, differentially expressed genes; Log2FC, base‐2 logarithm of fold change; TPM, transcripts per million.

We propose a model for suppressing anthocyanin accumulation in the MT‐*Aft/atv/hp2* mesocarp, where the pigmented epicarp creates a shading effect on the internal tissues of the fruit, thereby preventing the mesocarp from initiating anthocyanin biosynthesis. To evaluate this hypothesis, we examined the transcriptional level of photoreceptor genes: phytochromes (sensors of red and far‐red light: *SlPHYA*, *SlPHYB1*, and *SlPHYB2*), cryptochromes (sensors of blue/UV light: *SlCRY1a*, *SlCRY1b*, *SlCRY2*, and *SlCRY3*), and *SlUVR8* (a sensor of UV‐B light). In our study, *SlCRY3* (*Solyc08g074270*) showed a higher expression in the epicarp than the mesocarp of fruits developed under normal light conditions (Figure 4A; Table S7). Furthermore, *SlCRY3* expression was repressed in the epicarp of the fruit just uncovered (0d) compared to the control. In contrast, no difference was observed between the mesocarp at 0d and the control fruit (Figure 4A; Table S7). *SlCRY3* was upregulated in the mesocarp at 2d of light exposure compared to 0d, indicating that light reached the mesocarp and activated its expression at 2d in this tissue. On the other hand, in neither tissue, *SlCRY1a* (*Solyc04g074180*), *SlCRY1b* (*Solyc12g057040*), or *SlCRY2* (*Solyc09g090100*) showed differential expression between the epicarp and mesocarp of fruits developed under light (control), or when comparing the same tissue at 0d with the control conditions (Figure 4A; Table S7).

The UV‐B‐responsive photoreceptor gene *SlUVR8* (*Solyc05g018630*) showed higher expression in the epicarp compared to the mesocarp in the control conditions. Furthermore, it was upregulated in the mesocarp at 2d and 5d light exposure compared to the control mesocarp (Figure 4A; Table S7). The expression of genes coding for the phytochromes *SlPHYB1* (*Solyc01g059870*) and *SlPHYB2* (*Solyc05g053410*) was repressed in the epicarp control compared to the mesocarp control. In contrast, *SlPHYA* (*Solyc10g044670*) did not show a significant difference in this comparison (Figure 4A; Table S7).

### 3.5 Analysis of Anthocyanin‐Related Transcription Factor Gene Expression Patterns in MT‐*Aft/atv/hp2* Tomato Fruit Tissues

**FIGURE 5:**
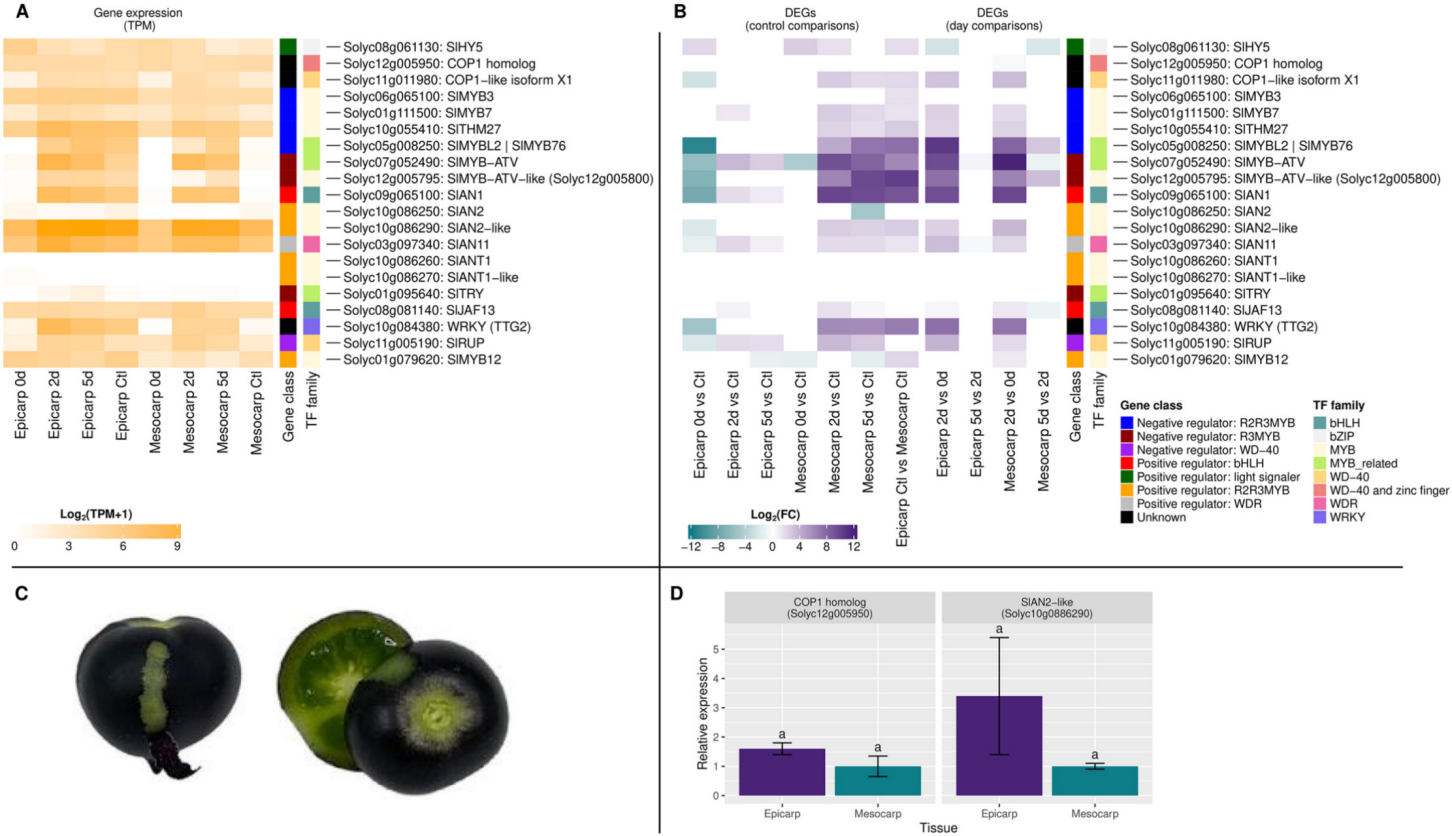
Transcriptional level of the anthocyanin biosynthetic regulatory genes in tissues of tomato fruits (MT‐*Aft/atv/hp2*). (A) Expression [log2 (TPM + 1)] and (B) differential expression (log2FC) of the anthocyanin biosynthetic regulatory genes in the epicarp and mesocarp of tomato fruits (MT‐*Aft/atv/hp2*) exposed to different light conditions. (C) MT‐*Aft/atv/hp2* at the mature green stage of development. (D) RT‐qPCR analysis of *SlAN2‐like* (*Solyc10g086290*) and *COP1 homolog* (*Solyc12g005950*) genes was performed in fruit tissues (epicarp and mesocarp) of MT‐*Aft/atv/hp2* at the mature green stage of development. The fruits were developed under normal light conditions. Data is means of three biological replicates. The same letters indicate no significant differences at *p* ≤ 0.05. Abbreviations: Ctl, control; d, days of light exposure; DEGs, differentially expressed genes; Log2FC, base‐2 logarithm of fold change, TPM, transcripts per million.

We analyzed the expression of some transcription factors that act upstream of the MBW complex in anthocyanin biosynthesis activation (Figure 5A; Table S8). The expression of the *SlHY5* (*Solyc08g061130*) gene was upregulated in the epicarp at 0d light exposure and in the mesocarp at 0d and 2d compared to the control tissues of fruits developed under normal light conditions. Interestingly, *SlHY5* expression was induced in the acyanic epicarp developed without light (0d) compared to the cyanic epicarp (control). The transcription factor *SlWRKY* (*Solyc10g084380*) was repressed in the epicarp at 0d light exposure compared to the cyanic fruit control. In contrast, it was induced in the mesocarp at 2d and 5d light exposure compared to the acyanic mesocarp of the control fruits. Furthermore, *SlWRKY* was induced in the epicarp compared to the mesocarp in control conditions. This expression pattern aligns with the development of anthocyanin pigmentation observed in the MT‐*Aft/atv/hp2* fruit tissues. We also observed that the transcriptional levels of *CONSTITUTIVE PHOTOMORPHOGENIC 1* (*COP1 homolog* [*Solyc12g005950*] and *COP1‐like isoform X1* [*Solycg11g011980*]) were similar across the analyzed conditions, except for the epicarp and mesocarp at 0d, where the *COP1‐like isoform X1* was expressed at lower levels (Figure 5A; Table S8).

Among the MYB TF genes, *SlANT1* (*Solyc10g086260*) and *SlANT1‐like* (*Solyc10g086270*) were not expressed in any of the tissues or treatments analyzed, except for the epicarp at 0d, where *SlANT1‐like* showed a very low value (Figure 5A; Table S8). The *SlAN2* (*Solyc10g086250*) showed very few transcripts in the epicarp and mesocarp in all conditions analyzed. By contrast, *SlAN2‐like* (*Solyc10g086290*) showed high expression levels in all tissues and conditions analyzed. In the epicarp, there was no differential expression of the *SlAN2‐like* at 2d and 5d light exposure, while it was downregulated at 0d compared to the light‐exposed control. *SlAN2‐like* expression was induced at 2d and 5d in the mesocarp but was not differentially expressed at 0d compared to the control (Figure 5A,B; Table S8). Furthermore, the RT‐qPCR confirmed the slight difference between the *SlAN2‐like* expression in the anthocyanin‐pigmented epicarp and the non‐pigmented mesocarp (Figure 5C,D).

SlAN2‐like interacts with the constituent factors bHLH1 (SlJAF13) and WDR (SlAN11) to form the first MBW complex (Chaves‐Silva et al. 2018). In our study, *SlJAF13* (*Solyc08g081140*) and *SlAN11* (*Solyc03g097340*) were expressed in all cyanic and acyanic tissues analyzed (Figure 5A; Table S8). In the epicarp, *SlJAF13* expression was induced at 2d light exposure, whereas *SlAN11* expression was lower at 0d but higher at 2d and 5d when compared to the light‐exposed control. In the mesocarp, *SlJAF13* and *SlAN11* expression levels were not different at 0d compared to the control, but they were induced at 2d and 5d. Subsequently, the formation of the first MBW complex induces the expression of *bHLH2* (*SlAN1*: *Solyc09g065100*). This, in turn, replaces bHLH1 and leads to the assembly of the second MBW complex, ultimately activating the anthocyanin structural genes. In both tissues, *SlAN1* expression was minimal at 0d light exposure, whereas it highly increased at 2d and 5d (Figure 5B; Table S8), which correlates with anthocyanin pigmentation in tomato fruit tissues.

### 3.6 Expression of Specific MYB Regulators of Anthocyanin Biosynthesis in MT‐*Aft/atv/hp2* Tomato Fruit Tissues

We examined the role of the second MBW complex in activating the expression of the negative anthocyanin regulators: *SlMYB‐ATV* (*Solyc07g052490*), *SlMYB‐ATV‐like* (*Solyc12g005795*), *SlTRY* (*Solyc01g095640*), *SlMYB3* (*Solyc06g065100*), *SlMYB7* (*Solyc01g111500*), *SlMYB32* (*THM27*—*Solyc10g055410*), and *SlMYBL2/SlMYB76* (*Solyc05g008250*). Although these MYB repressors were expressed in all samples (Figure 5A; Table S8), the *SlMYB‐ATV* expression pattern matches that of *SlAN1*, suggesting that the second MBW complex coordinates the transcription of both genes.

### 3.7 Expression of Structural Genes Associated With Anthocyanin Biosynthesis in Purple Fruits

**FIGURE 6:**
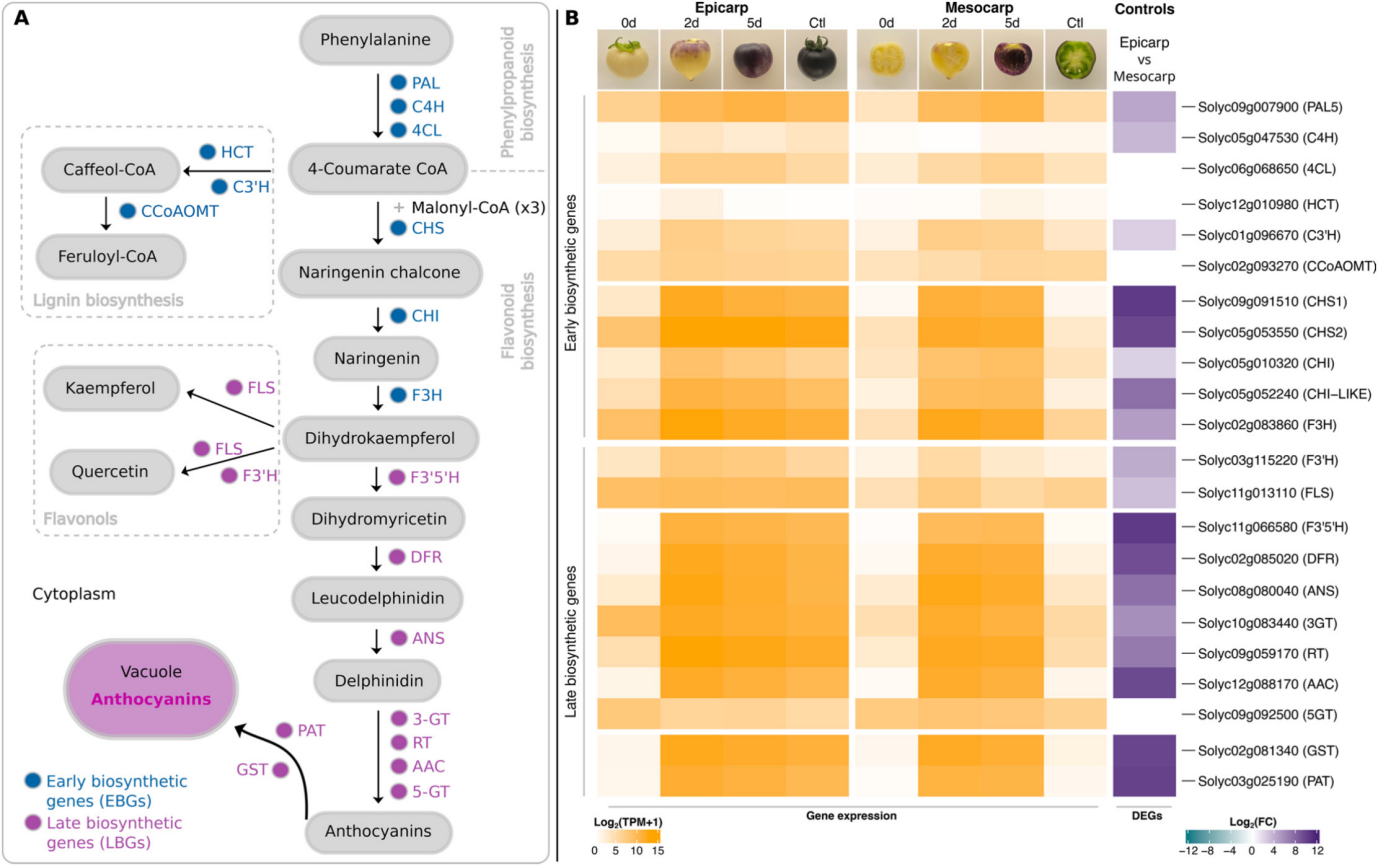
Anthocyanin biosynthesis pathway and expression patterns of the early biosynthetic genes and late biosynthetic genes in tissues of tomato fruits (MT‐*Aft/atv/hp2*). (A) Anthocyanin biosynthesis pathway. Adapted from Qiu et al. (2019). (B) Expression [log2 (TPM + 1)] and differential gene expression (log2 FC) of the anthocyanin Early biosynthetic genes and Late biosynthetic genes in the epicarp and mesocarp of purple tomato fruits (MT‐*Aft/atv/hp2*). Abbreviations: Ctl, control; d, days; Log2 FC, base‐2 logarithm of fold change; TPM, transcripts per million reads.

Regulatory and structural genes regulate the anthocyanin biosynthesis pathway (Figure 6A). In the epicarp, the expression of almost all structural genes was highly downregulated at 0d of light exposure, compared with the control. In contrast, at 2d and 5d of light exposure, their expression levels were similar to the control (Figure 6B; Table S9). Interestingly, in the mesocarp at 2d and 5d light exposure conditions, EBGs and LBGs were highly upregulated compared with the acyanic mesocarp control developed under normal light conditions, matching the anthocyanin accumulation in the fruit tissues (Figure 6B; Table S9).

## 4 Discussion

### 4.1 Light‐Dependent Anthocyanin Biosynthesis Activation in the MT‐*Aft/atv/hp2* Fruits

Genetic and environmental parameters directly influence the anthocyanin biosynthesis pathway in the tomato fruit (Albert et al. 2014). Light‐mediated signals are usually essential to activating this pathway in different tissues (Liu et al. 2018). In addition, fruit development also influences anthocyanin accumulation, with some anthocyanin‐enriched tomato genotypes only starting to accumulate this compound after a specific stage (Qiu et al. 2019; Sun et al. 2020). Our cyanic genotype MT‐*Aft/atv/hp2* is an anthocyanin‐enriched tomato line that accumulates anthocyanins in the subepidermal layer of the epicarp (the peel), thus developing dark purple fruits. This line was developed by introgressing natural genetic variation from two wild species and a cultivar of tomato (the loci *Aft*, *atv*, and *hp2*) to create a near‐isogenic line (NIL) in the cv. Micro‐Tom background: the MT‐*Aft/atv/hp2* (Sestari et al. 2014).

Fruits of tomato lines containing both alleles *Aft* and *atv* in homozygosity in cv. Indigo Rose showed progressive accumulation of anthocyanins, starting right before the mature green stage on the side directly exposed to light, whereas the shaded side remained green at this stage (Qiu et al. 2019; Sun et al. 2020). On the other hand, MT‐*Aft/atv/hp2* fruits started accumulating anthocyanins in the epicarp right after petal senescence (Figure 1A). The difference in pigmentation pattern is due to the *hp2* allele, a loss of *DEETIOLATED* (*DET1*) function, a negative regulator of light signal transduction, conferring hypersensitivity to light (Levin et al. 2003). However, when growing under regular light exposure, anthocyanin accumulation in MT‐*Aft/atv/hp2* fruits remained restricted to the epicarp. In contrast, the mesocarp and the region under the sepals remained acyanic (Figure 1C). This pattern shows that light is essential to activating anthocyanin biosynthesis in the purple tomato fruits.

### 4.2 Anthocyanin Pigmentation Patterns Correlate With Light Exposure

Anthocyanin accumulation is commonly restricted to the subepidermal cells and absent in the parenchymal cells of the mesocarp, mesophyll, and cortex. Substrate is likely to be available in these cells since the expression of specific transgenic MYB and bHLH transcription factors leads to high anthocyanin accumulation in the inner tissues of the tomato fruits (Butelli et al. 2008; Cerqueira et al. 2023). Therefore, other mechanisms should explain this “parenchymal recalcitrance”, a widespread phenomenon throughout angiosperms (Chaves‐Silva et al. 2018).

Here, we demonstrated that the restriction of light incidence over MT‐*Aft/atv/hp2* fruits completely inhibited the anthocyanin pigmentation in all tissues (Figure 2). Similar results of the anthocyanin pigmentation inhibition in the dark were observed in tomato (Xu et al. 2022), apple (Li et al. 2012), broccoli (Liu et al. 2020), chrysanthemum (Hong et al. 2015), and eggplant (Jiang et al. 2016; Li et al. 2024), confirming that light is a major factor controlling anthocyanin biosynthesis (Liu et al. 2018). Subsequently, 5 days of light exposure of the non‐pigmented, mature green MT‐*Aft/atv/hp2* fruits, grown in the dark for 30 days, to light conditions, led to rapid activation of the anthocyanin biosynthesis in both the epicarp and mesocarp (Figure 2). This observation shows that the activation of the anthocyanin biosynthesis pathway in the MT‐*Aft/atv/hp2* fruit depends directly on the incidence of light and is developmentally independent. Moreover, although some studies have successfully obtained anthocyanin accumulation in the mesocarp via transgenic methods (Butelli et al. 2008; Sun et al. 2020), our study is the first to report anthocyanin pigmentation in the tomato mesocarp through natural genetic variation. These findings led us to reason that the pigmented epicarp of MT‐*Aft/atv/hp2* acted as a light‐blocking layer starting in the first stage of fruit development, thus preventing light from reaching the mesocarp. Our experimental design, therefore, allowed the inhibition of the early pigmentation of the epicarp and facilitated the penetration of light into the non‐pigmented epicarp, triggering anthocyanin biosynthesis in the mesocarp (Figure S3).

### 4.3 Photoreceptors Involved in the Anthocyanin Biosynthesis Activation in Tomato Fruits

Plants use specific photoreceptor classes to receive light signals and coordinate stimulus responses. The cryptochrome *CRY3* participates in anthocyanin biosynthesis in eggplant, purple broccoli, and petunia. *CRY3* expression is repressed during shading conditions and highly induced by light (Li et al. 2017; Fu et al. 2020; Liu et al. 2020). In petunia, while *CRY3* was repressed by exposure to red light compared to white and blue lights, *CRY1* and *CRY2* did not show significant changes in response to the light quality (Fu et al. 2020). The synergistic effect of blue and UV‐B light promoted anthocyanin accumulation in the epicarp of the tomato *Aft* line (Kim et al. 2021). In our study, *SlCRY3* and *SlUVR8* were the only photoreceptors transcriptionally induced in the cyanic epicarp compared with the acyanic mesocarp in control conditions. *SlUVR8* expression increased in both tissues along the days of light exposure for the fruits grown in the dark, whereas *SlCRY3* peaked at 2d when anthocyanin accumulation started becoming noticeable (Figure 4B). Based on these gene expression patterns and the current understanding that short‐wavelength radiation (i.e., blue and UV lights) promotes anthocyanin accumulation, we infer that *SlCRY3* and *SlUVR8* are the primary photoreceptors activating the anthocyanin pigmentation in tomato fruits.

Therefore, the anthocyanin pigmentation of the MT‐*Aft/atv/hp2* epicarp starts at the first stage of fruit development and directly influences the quantity and quality of light in the mesocarp by blocking short‐wavelength radiation. This light‐blocking effect leads to the inactivation of light‐induced signal transduction in the mesocarp, which is necessary to activate anthocyanin biosynthesis‐responsive genes (Figure S3).

The *SlRUP* gene functions as a negative regulator of UV‐B light signaling responses by promoting the reversion of the active *SlUVR8* monomer into its inactive homodimer form, thereby modulating photomorphogenic responses (Zhang et al. 2021). Given that the expression levels of SlUVR8 identified in this study suggest its role as a key regulator of the anthocyanin biosynthesis pathway, *SlRUP* may act as a potential negative regulator of this pathway due to its repressive effect on SlUVR8. In our study, the expression levels of *SlRUP* were responsive to light (Figure 5; Table S8), consistent with the observations in the Ailsa Craig genotype developed under light conditions (Zhang et al. 2021). Apparently, in the MT‐*Aft/atv/hp2* genotype, the presence of *SlRUP* was not enough to influence the lack of anthocyanin accumulation in fruit tissues. However, future investigations into the role of this gene in regulating *SlUVR8* conformation, particularly anthocyanin‐enriched genotypes such as MT‐*Aft/atv/hp2*, may contribute to enhancing the accumulation of this compound in fruit tissues. Furthermore, it is important to explore the stability of *SlRUP* in these genotypes, considering they carry alleles from wild species, which may have led to structural alterations in *SlRUP* that affect its ability to redimerize *SlUVR8*.

### 4.4 Transcriptional Patterns of Anthocyanin‐Positive Regulatory Genes May Explain the Lack of Anthocyanin Synthesis in the Mesocarp

We demonstrated that anthocyanin accumulation in MT‐*Aft*/*atv*/*hp2* fruits is light‐dependent. Tomato lines with the dominant *Aft* (*Anthocyanin fruit*) allele display a spotted‐purple phenotype linked to a genomic region that contains four in‐tandem R2R3 MYB genes (Sapir et al. 2008; Cao et al. 2017): *SlAN2* (*Solyc10g086250*), *SlANT1* (*Solyc10g086260*), *SlANT1‐like* (*Solyc10g086270*), and *SlAN2‐like* (*Solyc10g086290*), which corresponds to the *AFT* gene. Figure 5A shows that *SlANT1*, *SlANT1‐like*, and *SlAN2* displayed no or very low expression levels in both cyanic and acyanic fruit tissues. Similar studies on tomato genotypes with the *Aft* locus found insignificant expression levels for these genes (Qiu et al. 2019; Colanero, Tagliani, et al. 2020; Sun et al. 2020), showing that they are not involved in regulating anthocyanin biosynthesis in *Aft*‐bearing purple tomato fruits.

In contrast, *SlAN2‐like* was highly expressed in all tissues and conditions analyzed. Its expression was only slightly lower in the non‐pigmented epicarp at 0d of exposure to light compared to the purple epicarp of fruits developed under normal light conditions (Figure 5B). The same pattern was reported for the “Indigo Rose” cultivar and a mutant with reduced anthocyanin pigmentation (Qiu et al. 2019).

The MYB transcription factor SlAN2‐like interacts with the constitutive factors bHLH1 (SlJAF13) and WDR (SlAN11) to form the first MBW complex, which induces *bHLH2* (*SlAN1*) expression. Subsequently, bHLH2 (SlAN1) replaces bHLH1 (SlJAF13) to configure the second MBW complex. This second complex, in turn, activates the expression of *SlAN1* (“reinforcement mechanism”) and the LBGs (Colanero, Tagliani, et al. 2020). Even though *SlJAF13* and *SlAN11* expression levels fluctuated across samples, they were constitutively detected in pigmented and non‐pigmented tissues (Figure 5A), which was also observed in other studies (Gao et al. 2018; Gonzali et al. 2025). Transcriptional analysis in different purple tomato lines showed that *SlAN1* expression is correlated with the level of anthocyanin pigmentation (Butelli et al. 2008; Bassolino et al. 2013; Qiu et al. 2016, 2019; Gonzali et al. 2025), which was confirmed in our study (Figure 5A). Our findings indicate that *SlAN1* may be the limiting factor governing the development of anthocyanin pigmentation of tomato fruit tissues, with its expression being light‐dependent.

Although *SlAN2‐like* was the most highly expressed anthocyanin‐related MYB factor across all tissues and conditions analyzed, its expression levels remained relatively stable in both cyanic and non‐cyanic tissues of tomato fruits at 30–35 days after anthesis (Figure 5A). These findings led us to speculate about a possible negative regulation of the SlAN2‐like protein by COP1 in tomato fruit tissues under dark conditions, similar to what is observed in apples and eggplants (Li et al. 2012, 2024).

Transcription factors from the WRKY family are essential regulators of anthocyanin biosynthesis in an HY5‐independent manner (Qiu et al. 2019). WRKY physically interacts with WD to form a transcriptional complex independent of the MBW complex, leading to the transcriptional activation of membrane transporter and vacuolar acidification genes (Lloyd et al. 2017). In our study, the *SlWRKY* transcriptional profile matches the anthocyanin accumulation pattern in MT‐*Aft/atv/hp2* fruit tissues (Figure 5A), which was also observed by Gonzali et al. 2025. Thus, our model suggests that *SlWRKY* expression is regulated by light and that it plays an essential cyanogenic role in tomato fruits by activating the transcription of anthocyanin structural genes.

### 4.5 COP1 May Act as a Negative Regulator of SlAN2‐Like Activity in Tomato Fruit Tissues Developed in the Dark

The interaction between COP1 and MYB proteins in regulating anthocyanin accumulation has been observed in both apple and eggplants. In apple, MdCOP1 interacts with MdMYB1 to regulate light‐induced anthocyanin biosynthesis (Li et al. 2012), while in eggplants, SmCOP1 interacts with SmMYB5, promoting its degradation via the 26S proteasome pathway (Li et al. 2024). The gene expression patterns of *SlCRY3*, *SlUVR8*, *COP1 homolog*, *COP1‐like isoform X1*, and *SlAN2‐like* observed in this study (Figures 4B and 5) suggest that similar interactions may also occur in tomato fruits, directly influencing anthocyanin accumulation.

Both *COP1* genes, the *COP1 homolog* and the *COP1‐like isoform X1*, showed substantial expression levels in the epicarp and mesocarp tissues of the MT‐*Aft/atv/hp2* genotype under the conditions analyzed in this study. Additionally, Menconi et al. (2025) reported expression of both genes in the MT‐*Aft/atv/hp2* and MT‐*Aft/atv* genotypes, along with evidence of protein activity in protoplasts. These findings indicate that both genes have the potential to function as COP1 in tomato plants. Nevertheless, further functional analyses are required to determine which of the two plays the dominant role in protein degradation under dark conditions in tomato fruit tissues.

**FIGURE 7:**
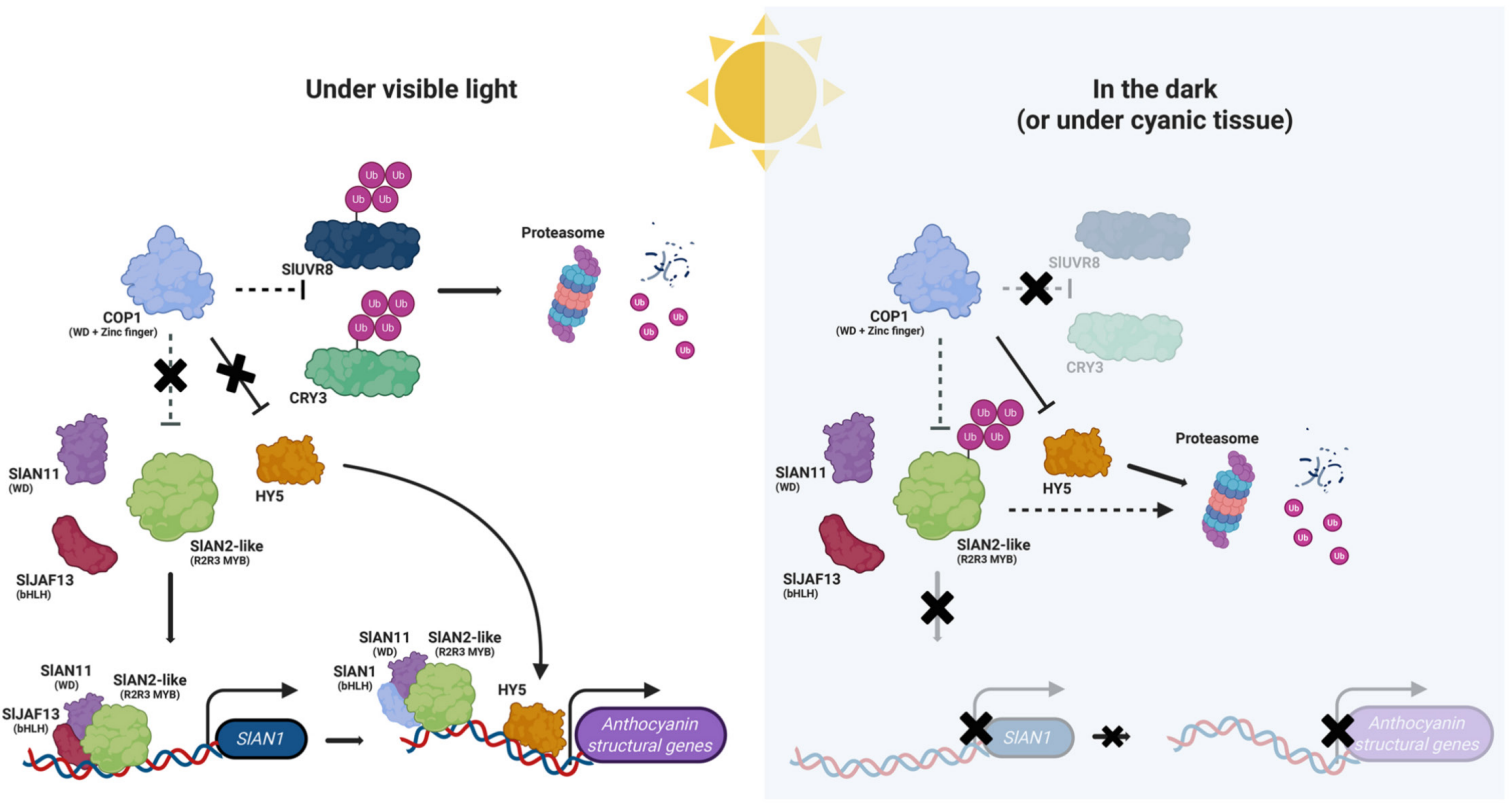
Possible transcriptional model for anthocyanin biosynthesis regulation under light and dark conditions. Under visible light, instead of ubiquitinating SlAN2‐like (*Solyc10g086290*), COP1 (*Solyc12g005950*) ubiquitinates the photoreceptors SlUVR8 (*Solyc05g018630*) and CRY3 (*Solyc08g074270*), leading to their degradation by the proteasome. In this condition, SlAN2‐like forms the first MBW complex together with SlJAF13 (*Solyc08g081140*) and SlAN11 (*Solyc03g097340*) to activate SlAN1 (*Solyc09g065100*) gene expression. After that, SlAN1 replaces SlJAF13 to form the second MBW complex to activate the anthocyanin structural genes. In dark conditions, COP1 will ubiquitinate SlAN2‐like, inducing its degradation by the proteasome, inhibiting the formation of the first MBW complex by activating *SlAN1* expression and, consequently, the anthocyanin biosynthesis.

Despite the high expression of *SlAN2‐like* under dark conditions (acyanic tissues; Figure 5A), its activity might be post‐translationally suppressed by COP1. Under light conditions, however, the transcription of *SlCRY3* and *SlUVR8* is upregulated (Figure 4B), indicating that these photoreceptors may become the primary targets of COP1, allowing SlAN2‐like to escape repression. In summary, under dark conditions, COP1 likely functions as a negative regulator of the SlAN2‐like protein, preventing the assembly of the first MBW complex and, consequently, inhibiting *SlAN1* expression. Conversely, light triggers the expression of photoreceptor genes, redirecting COP1 activity toward these newly available targets. This redirection allows the formation of the first MBW complex (SlAN2‐like/SlJAF13/SlAN11), ultimately activating *SlAN1* expression (Figure 7).

Although the *det1* mutation, present in *hp2* genotypes, compromises the functional integrity of the COP1‐mediated protein complex (Cañibano et al. 2021), the possibility that this system retains some residual activity in some genes cannot be ruled out. It is important to note that this knowledge is derived from studies conducted in *Arabidopsis thaliana* , and its direct extrapolation to *S. lycopersicum* should be approached with caution, given the differences in the composition and regulation of COP1 and DET1 factors between species. Furthermore, our GO term analysis revealed an enrichment of protein ubiquitination (GO:0016567) at day 0 in the acyanic epicarp, compared to fruits exposed to normal light, as well as in the epicarp compared to the mesocarp of control samples (Figure S5). Therefore, based on the transcriptional profile of the genes analyzed here and the anthocyanin pigmentation pattern, our hypothesis is promising and warrants further investigation, including direct experimental validation at the protein level.

### 4.6 Structural Genes and MYB Repressor Expression in the MT‐*Aft/atv/hp2* Genotype

Genes that encode enzymes of the anthocyanin pathway are divided into “early biosynthetic genes” (EBGs) and “late biosynthetic genes” (LBGs; Quattrocchio et al. 2006). EBGs are related to synthesizing flavonoid precursors and final products, e.g., chalcones, dihydroflavonols, and flavonols. In contrast, LBGs are more specific for anthocyanins (Figure 6A). In our study, the expression pattern of anthocyanin structural and regulatory genes correlated with anthocyanin pigmentation in the tissues of tomato fruits (Figure 6B), the same expression pattern observed by Gonzali et al. (2025).

In addition to activating *SlAN1* and LBGs, the second MBW complex also induces the expression of MYB repressors (Albert et al. 2014; Colanero, Tagliani, et al. 2020). The R3‐MYB protein can directly bind to the bHLH factors, inhibiting the formation of the MBW complex activator of anthocyanin‐related genes (Colanero et al. 2018; Sun et al. 2020).

*SlMYB‐ATV* (*Solyc07g052490*) inhibits anthocyanin biosynthesis in tomato fruits (Cao et al. 2017; Colanero et al. 2018). Its transcriptional activation is triggered by the MBW ternary complex (SlAN2‐like/SlAN1/SlAN11), which also promotes *SlAN1* expression (Colanero et al. 2018). In our study, the expression pattern of *SlMYB‐ATV* mirrors that of *SlAN1* (Figure 5A), indicating that both genes are activated by the same complex. The functional, dominant *SlMYB‐ATV* allele exists in *S. lycopersicum* , while the recessive *atv* allele is found in some wild species, such as *S. cheesmaniae* (Cao et al. 2017). The *atv* allele produces a truncated, non‐functional protein due to a 4‐bp insertion in the second exon, causing a frameshift and a premature stop codon. This results in a truncated SlMYB‐ATV protein lacking the R3‐domain, which cannot bind bHLH factors and thus cannot disrupt the MBW complex (Cao et al. 2017; Colanero et al. 2018; Sun et al. 2020), leading to increased anthocyanin levels in tomato tissues. Therefore, the nonfunctional *SlMYB‐ATV* allele promotes anthocyanin pigmentation in tomato fruit tissues.

### 4.7 Additional Traits Observed in MT‐*Aft/atv/hp2* Plants

The yield of MT‐*Aft/atv/hp2* plants was reported to be comparable to that of cv. MT. MT‐*Aft/atv/hp2* fruits showed significantly higher levels of ascorbic acid, lycopene, and β‐carotene than cv. MT (Sestari et al. 2014). Anthocyanins have also been associated with increased resistance to biotic stresses in mango (Sivankalyani et al. 2016). We observed that MT‐*Aft/atv/hp2* plants appeared more resistant to thrips (*Thysanoptera*) than MT plants in greenhouse conditions. Even though we have not addressed this question in our current research, it warrants further investigation.

## 5 Conclusions

This study brings novel information on anthocyanin metabolism in the mesocarp cells of purple tomato fruits mediated by light. Short wavelengths, such as blue and UV‐B light, represent crucial signals for anthocyanin biosynthesis activation in cyanic tomato fruits. In the epicarp of MT‐*Aft/atv/hp2* fruits, these wavelengths are detected early during development and trigger signal transduction pathways, thereby inducing the expression of the anthocyanin regulatory and structural genes, resulting in anthocyanin accumulation. This pigmentation establishes a filter that blocks the short wavelengths from reaching the deep tissues of the fruit, consequently suppressing the expression of *SlAN1*, a likely limiting gene for anthocyanin accumulation. Exposing acyanic fruits, which develop in the dark, to light for 5 days promotes anthocyanin accumulation in the epicarp and the mesocarp tissues. Therefore, our results strengthen a working hypothesis to elucidate the anthocyanin recalcitrance observed in parenchymatic cells of inner fruit tissues. To overcome this resistance, we propose a reliable approach or genetic pathway preventing early epidermis pigmentation.

## Author Contributions

G.L.R., A.L.S., L.E.P.P., A.C.‐J., and V.A.B. conceived and planned the study. G.L.R., A.L.S., and V.A.B. designed the experiments. G.L.R., S.C.‐S., and A.L.S. performed the experiments. G.L.R., C.F.V., L.W.P.A., and V.A.B. analyzed the data. G.L.R., C.F.V., and L.W.P.A. wrote the manuscript. V.A.B., A.Z., L.E.P.P., and A.C.‐J. revised the manuscript. V.A.B. supervised all steps of the study and final manuscript. All authors read and approved the manuscript.

## Funding

This work is partly supported by the USDA National Institute of Food and Agriculture, Hatch project 11400036 (WVA00754). The Brazilian funding agency Coordination for the Improvement of Higher Education Personnel (CAPES) provided scholarships to G.L.R. under fellowship 88887.642843/2021‐00. The “Conselho Nacional de Desenvolvimento Científico e Tecnológico” (CNPq, Brazil) provided research scholarships to A.C.‐J., L.E.P.P., and A.Z. under grant numbers 309005/2022‐1, 308701/2022‐4, and 445056/2024‐0, respectively.

## Conflicts of Interest

The authors declare no conflicts of interest.

## Data Availability

The RNA‐seq data underlying this article are available in the Gene Expression Omnibus (GEO) Database (GSE235565). Scripts used for differential gene expression, and other analyses can be found in GitHub repository: https://github.com/Luis‐WP‐Arge/purple_tomato_RNA‐seq.
